# Progress in Ophthalmology

**Published:** 1898-01-15

**Authors:** 


### PROGRESS IN OPHTHALMOLOGY.

**Toxic Amblyopia.**—De Schweinitz,[1] in reporting a case of toxic amblyopia in which he had the opportunity of making a post-mortem examination owing to the death of the patient from pneumonia coupled with albuminuria, makes some interesting observations, mainly strengthening the views held formerly as to the situation and nature of the lesion in the optic nerve. He says: "The degeneration has attacked that portion of the optic nerve ordinarily known as the papillo macular bundle. It pursues a course similar to the one already described by Samelsohn, Vossius Uhthoff, and other observers, beginning in a wedge-shaped area on the temporal side of the optic nerve, which position it maintains, though in somewhat altered shape, being at first heart-shaped, and later crescentic for about 10 m.m. behind the globe. The area then approaches, but never reaches, the centre of the nerve, and maintains this position till it reaches the foramen opticum. In the intra-canial portion of the nerve the patch again becomes distinctly crescentic, and occupies a position above the centre. In the chiasm the foci of degeneration are symmetrically placed slightly below the centre, while in the tract the position is almost exactly central. He found, furthermore, that the degeneration was more pronounced on the right than the left side, which corresponded to the visual field examination, the right eye showing a larger scotoma and greater oss of colour perception, with spots of absolute scotoma. The degeneration on both sides was more marked just behind the lamina cribosa, and again at the optic foramen. As to the condition of the retina, he found the nerve fibre layer narrower than normal, and the ganglion cells in the neighbourhood of the nerve head to be smaller, and with an imperfect development of their processes, which phenomenon had been previously noticed by Sachs. In the macular zone, however, these cells showed neither atrophy nor degeneration. The coats of the retinal vessels were greatly thickened, which accorded with the arterial change elsewhere in the ocular tissues. The chorioid was free from change except those referred to in the vessels. In the optic nerves, as the site of the lesion is approached, there was an augmentation of the neuclei— a marked thickening of the connective tissue septa and a disappearance and degeneration of the nerve fibres. The diseased patch was sharply separated from its normal surroundings. The small nutrient vessels of the optic nerves were great y thickened, in some cases so much so that they resembled small fibrous cords. As to the actual pathological cause of toxic amblyopia, De Schweinitz says, we do not know, but that the vascular disturbances theory, mentioned by Sachs, has a good deal to recommend it and, seems borne out by the action of other toxic agents. De Schweinitz maintains that tobacco alone, or its active principle nicotine, is not the essential poisonous agent, but that one or more of the many principles freely present in tobacco-smoke, or some toxic influence which they liberate in the system, must be held accountable for the disease.

**Cautery in Corneal Ulceration.**—Rodman,[2] of Waterbury, Conn., is very sanguine about the use of the cautery in corneal ulceration. He quotes three cases in all of which the ulceration was of long standing, and says that the "converting of a corneal ulcer into a corneal burn" is followed by the best results. He advocates the galvano cautery, the spring making the connection being worked by the foot which allows of a more delicate touch, and sums up his observation by saying—to cure quickly and surely, cauterise. Unless inevitable from previous destruction of tissues opacities do not result. Heat radiation is minimised by the galvano cautery and rheostat. Cocaine anæsthesia is perfect and applicable unless in the case of young children. With the electrode in contact with the cocanised cornea before the electric current is completed a cauterisation can be effected without the knowledge of the patient.

**Tubercle of the Iris.**—In reporting a case of tubercle of the iris, Joseph Andrew[3], of New York, gives a most exhaustive *résumé* of all the reported cases of this disease—going back to 1868. He quotes 30 cases, and from these draws his conclusions that tubercle does not primarily occur in the iris. As to the diagnostic sign he says the growth consists of one or more nodules varying from the size of a pin's head to that of a pea, or even larger, the colour being light yellowish-white, light greyish-white or light greyish-yellow, whereas in sarcoma of ir s the colour is reddish-grey, blackish, light brown, or flesh-colour; and in a guonma the colour is an iron red or deep greyish red. Usually no vessels are seen on the surface of the tuberculous growth, there is little or no pain, and usually very little injection of the conjunctival vessels of the globe. The disease may begin as a serom or adhesive irido cyclitis. Tubercle is of more rapid growth than sarcoma of the iris.

As to prognosis he says: It appears from Leber's case that when the eye is enucleated, in the absence of other signs of tubercle elsewhere in the body, the prognosis is not unfavourable. In this case the patient was reported well after six years. As to treatment, when the tuberculous process is limited to a small area of the iris, does not encroach on the periphery, and there are no signs of tuberculous disease elsewhere, the patient should be given the benefit of the doubt as to iris being the primary seat of the disease and an iridectomy done including the growth in the portion of iris removed. When the disease is extensive and involves the ciliary body, and other signs of tuberculosis are absent, the eye should be enucleated.

**Functions of the Rods and Cones.**—Pratt,[4] in the *Medical Record*, goes extensively into the functions of the rods and cones in the retina, and comes to the conclusion that the cones are the only sensitive parts, the rods being m rely for their support. He quotes Hosch as having demonstrated by Golgi's silver staining the existence of direct anatomical continuity between the fibrils proceeding from the base of the cone cell and the processes of the nerve cell, and says no such continuity has been demonstrated or claimed for the rod.

[1] American Journal of Medical Sciences, Sept., 1897. [2] The Medical Record, Aug., 1897. [3] International Medical Magazine, Aug., 1897. [4] Medical Record, Aug. 28, 1897.

